# Spotted hyaena *Crocuta crocuta* feeding ecology and selectivity of large herbivorous prey in the Namib desert

**DOI:** 10.1002/ece3.7302

**Published:** 2021-03-11

**Authors:** Karl Sebastian Moritz Fester, Georgina Hockings, Rudie Jansen van Vuuren, Marlice van Vuuren

**Affiliations:** ^1^ Kanaan N/a’an ku sê Desert Retreat Windhoek Namibia; ^2^ N/a’an ku sê Foundation NWE Windhoek Namibia

**Keywords:** *Crocuta crocuta*, feeding ecology, prey selection, spotted hyaena

## Abstract

We have investigated the relationship between spotted hyaenas in the south Namib Desert and large herbivorous prey and have summarized an updated overview of predator‐prey relationships in this resource‐limited arid environment. Over the 52‐month study, we recorded the densities (#/km^−2^, ±*SE*) of the four local large herbivorous prey species: gemsbok (1.229, ±0.50), springbok (1.352, ±0.48), ostrich (0.648, ±0.23), and greater kudu (0.343, ±0.00). A fecal analysis was performed on 146 collected spotted hyaena scats, and prey items were identified and hairs cross‐follicle analyzed to the species level. Spotted hyaena diet at the study area remained opportunistic with 240 identified prey items representing eight differing prey species being recorded, ranging from ostrich eggs to large ungulates. The Ivlev's Electivity Index was used to determine which large herbivorous prey was most selected for. Although gemsbok had a higher representation of prey items in the sampled scats, all sampled large herbivorous prey species scored below 0 and are thus generally avoided in relation to their availability in the environment. If any prey preferences are expressed by spotted hyaena in the Namib, it can be presumed to be a nonsampled prey species. We therefore promote further detailed investigations into all other prey species present, and seasonal variations of prey densities and scat sampling, within the study environment.

## Graphical abstract

Over a 52 month period, this study recorded the densities of resident large herbivorous prey and collected spotted hyaena scat samples in the south Namib Desert. The data were evaluated for prey selectivity employing the Ivlev's Electivity Index. No large prey species were found to be selected for beyond their density and no large prey species preference was met for spotted hyaena.

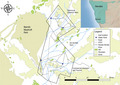

## 1 INTRODUCTION

Spotted hyaena (*Crocuta crocuta*, Erxleben) are large opportunistic apex carnivores able to hunt large‐bodied prey several times their own weight (Kruuk, 1972). Multiple studies have determined carnivore‐prey relationships across various biomes, leading to a general knowledge of spotted hyaena feeding ecology (Cusack et al., 2017). Their feeding relationships with both medium and large prey can influence population and spatial distributions through interactions within their geographical area (M'soka et al., 2016; Owen‐Smith & Mills, 2008). Studies have found that spotted hyaenas tend to persist in environments where they fluidly adjust their diets according to the most available prey (Cooper et al., 1999).

Spotted hyaena are present in the Namib‐Naukluft Park (NNP), an arid, resource‐limited environment within the south Namib Desert, Namibia (Mills & Hofer, 1998). Spotted hyaena diet of medium to large herbivorous prey through fecal analyses has been previously recorded in the Namib Desert by Tilson et al. (1980). Fecal analyses are widely accepted to help determine which prey species are consumed, and their frequency, by spotted hyaenas (Bearder, 1977), and where direct feeding instances are difficult or not observed (Rduch, 2016; Wentworth et al., 2011). It was also determined that spotted hyaena predation does not pose a limiting factor to prey populations in the Namib Desert (Henschel & Tilson, 1988). However, a more updated look into the feeding ecology and predator‐prey relationships between Namib hyaenas and herbivorous prey is needed.

Because spotted hyaena are considered generalist predators of prey between 56 kg and 182 kg (Hayward, 2006), this 52 month study (2016–2020) aimed to investigate the feeding ecology between spotted hyaenas and large herbivorous prey in the south Namib Desert. This was done through a prey population census and a presence‐absence based fecal analysis to determine prey selectivity and spotted hyaena persistence within this environment. It was hypothesized that large herbivorous prey is an important food source, and the large prey species with the highest density would be the most numerous encountered prey items identified within spotted hyaena scat.

## 2 METHODS

### 2.1 Study area

This study was conducted within the boundaries of Farm Kanaan, a 352 km^2^ tourism destination in south‐west Namibia (S‐25°49′23.0, E016°07′32.0), owned by the N/a'an ku sê Foundation. Kanaan is a 10 year postlivestock use area, where livestock fences (185 km), gravel roads (255 km), and four artificial water points still exist. Surrounding land uses include tourism, livestock farming (communal and commercial) focusing on sheep, goats, and cattle, or a combination of both tourism and livestock farming (EIS of Namibia, 2016; Mendelsohn et al. 2002).

The study area shares a 7.3 km border along the north with a communal farming area (255.5 km^2^) utilizing cattle, sheep, and goats. The east border (22.7 km) is shared with a 10 year de‐stocked farm (258.4 km^2^) with no current intensive management. The south and south‐east (23.3 km) borders are shared with commercial properties (222 km^2^ and 229 km^2^) utilizing cattle, equines (horses & donkeys), and tourism. The 35 km western border is shared with the protected NNP where no intensive management or anthropogenic intrusions exist. Livestock densities per carrying capacities are listed as 0–20 kg/ha for the study area location by Mendelsohn, et al. (2002).

The study area is an open desert landscape dominated by the Tiras Mountains in the south sloping westwards into the Namib Desert dune belt, with rocky hills sporadically breaking up the landscape. The study area encompassed only a small portion of the Namib biome, and therefore differences in vegetation and substrate structure, which can create differing habitats for predator and prey species, did not occur and were not assessed. Wildlife species within the area are free roaming and not permanently confined within the borders of the study area, with old fences creating a minor obstacle to movements. Annual rainfall is estimated at 50–100 mm mostly falling between January and June (Mendelsohn et al. 2002). Rainfall on Kanaan during the study period (2016–2020) was recorded as 62 mm, 28 mm, 104 mm, 17 mm & 15.5 mm, respectively.

Gemsbok (*Oryx gazella* Linnaeus) are the most commonly encountered large mammal species within the study area. Springbok (*Antidorcus marsupialis* Zimmermann), ostrich (*Struthio camelus* Linnaeus), greater kudu (*Tragelaphus strepsiceros* Pallas), (henceforth referred to as kudu), and klipspringer (*Oreotragus oreotragus* Zimmermann) are also encountered in the study area. Three other sympatric large carnivores occur in the area: leopard (*Panthera pardus* Linnaeus), cheetah (*Acinonyx jubatus* Schreber), and occasional brown hyaena (*Parahyaena brunnea* Thunberg). Medium carnivores in the study area which share a feeding relationship with local spotted hyaena are black‐backed jackal (*Canis mesomelas* Schreber), (Mendelsohn et al. 2002; Stuart & Stuart, 2006).

### 2.2 Prey densities

**FIGURE 1:**
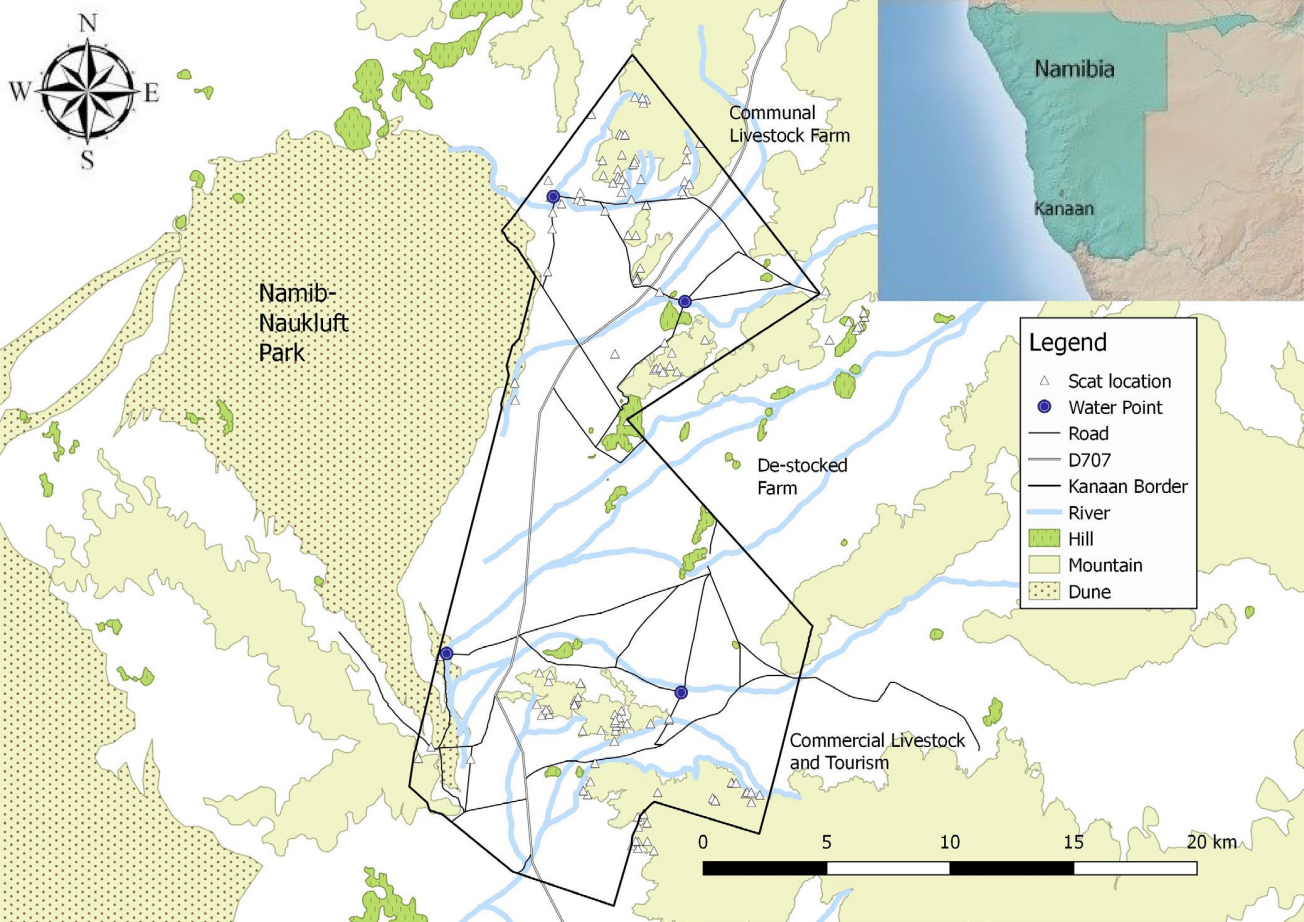
Map of the Kanaan study area, its location within Namibia (inset), and the locations of the 146 collected scats. Roads shown within the Kanaan border were used for the routine game census counts

Large herbivorous prey species densities were recorded by line‐transect game census counts (similar method employed by Buckland, 1985; Surendra Varman & Sukumar, 1995), performed twice weekly by open‐backed vehicle at a speed between 25–40 km/hr and for a consecutive duration not exceeding 2.5 hr. A total of 508 transects were driven at lengths between 23 and 34 km (mean = 28.96 km) using 100 m sighting increments with the maximum sighting distance limited to 1,500 m due to the openness of the environment and accuracy of measuring distances with a rangefinder. These transects used roads which traversed the entirety of the study area and allowed for sampling within all present landscapes and terrains (Figure 1).

Animal spotting was undertaken employing between five and ten volunteer observers per count, and only individuals or animal groupings within the borders of the study area were recorded. A perpendicular distance was recorded for each sighting by the two main authors on every census using a radial compass and a rangefinder. Due to a lack of game fences and confinement of the sampling area, means were calculated at the end of each sampling month for each species' group size and perpendicular distances, and a base species density per km^−2^ was estimated using Distance 7.3 software (Thomas et al. 2010) with parameters of the sampling area.

### 2.3 Spotted hyaena scat sampling

Spotted hyaena scats were collected between December 2017 and March 2020. A reference library of hair samples representing ten local wildlife species (gemsbok, kudu, springbok, klipspringer, black‐backed jackal, polecat, bat‐eared fox, yellow mongoose, aardwolf, and spotted hyaena) and five domestic species (cattle, sheep, goat, donkey, and horse) was created. Prey hair samples were opportunistically collected at the beginning of the study from local species' carcasses when found in the field, as well as provided by local owners of domestic livestock.

Hikes to locate spotted hyaena scat were conducted twice weekly with five to ten volunteer observers walking several meters apart side‐by‐side. Distances hiked were between three and twelve kilometers, with an average 500 m perpendicular viewing distance from ground level. Hikes were conducted from terrain or man‐made features toward open areas, or visa‐versa, as well as along the edges of terrain features and, when possible, following water course‐ways and game trails up into elevated terrain. These transects were conducted throughout the study area and were nonconsecutively rewalked at least three times during the study. When permission was given, these hikes were occasionally conducted on adjoining properties.

Spotted hyaena scats and latrine sites which were located either during the routine hikes or during game census counts were GPS marked and later GIS mapped (QGIS Development Team, 2009), measured, and recorded (Figure 1). Scats were collected in individual sealable plastic bags, dated, and numbered. A maximum of three scats were collected from latrine sites and selected depending on the varying states of decay to represent the diets over the chronological use of each site. Scats were collected across a range of colorations; greener coloration represented recent scats (within 2 weeks) while those bleached white through to the core represented older, oxidized scats (up to 1 year). The outer appearance of the scat (e.g., firm or crumbling outer appearance) was also taken into account in determining the age of the scat collected. Owing to a low presence and impact of *Trogidae* and *Scarabaeidae* beetles (Picker et al., 2002), scats of varying ages were available for sampling.

A more randomized approach to scat collection was adopted to minimize the impact on active latrines and anthropogenic disturbance which might alter temporal space use and behaviors of spotted hyaena (Belton et al., 2016; Green & Holekamp, 2019). Motion‐sensor cameras were placed at each latrine site to confirm the hyaena species using the latrine. Since spotted hyaena scats are differing slightly in size and spatial distribution within a latrine from brown hyaena (Skinner & van Aarde, 1981), differentiation between the two hyaenidae species could be determined. However, even with careful identification during collection through references (Stuart & Stuart, 2013), the possibility of misidentification of isolated scats outside of latrines between the two present hyaenidae species remained. This was accounted for by collecting only larger‐mass isolated scats and avoiding those scats found in the general vicinities of where brown hyaena had been seen on the motion‐sensor cameras.

Similar to procedures outlined by Mills and Mills (1978), once collected, samples were air dried for a period of 30 days, and then finely crushed to extract hairs and biological artifacts such as bone fragments, ostrich egg fragments, and feathers. Since ostrich eggs have been found to supplement the diets of several hyaenidae species across their ranges (Kruuk, 1972; Pienaar, 1969; Skinner & van Aarde, 1981), they were included under ostrich as a food item for this study. The crushed samples were then dissolved in clear water in fine‐mesh cloth to further clean and extract hair samples. Since remains of varying species are known to be represented in hyaena scat through collection within the hyaena's digestive tract, samples from all hairs of differing size, shape, and color were extracted from each dissolved sample (Rduch, 2016). Once dried, these samples were placed onto a microscope slide, numbered according to date and location, and secured under a cover slide. Similar to procedures incorporated by Maude and Mills (2005), a microscopic cross‐follicle analysis was performed by the first author against the hair reference library under a magnification of 160 (4/0.08–10/0.025) with an 8x eyepiece. Focus was given to the size and shape of the cuticle, and shape of the hair root during the cross‐follicle analysis. Identification to the species level of all collected prey items from the scat was attempted. Due to limitations in available equipment, gravimetric consistencies of prey items per scat were not recorded.

### 2.4 Prey selection

Ivlev's measure of electivity: (*Eᵢ* = (*rᵢ* − *nᵢ*)/(*rᵢ* + *nᵢ*)) was used to test for prey selectivity in relation to species abundance in the environment. Data were expressed as percentages and thus calculated as decimals. For Ivlev's Index, *r_i_* is the proportion of a prey species (*i*) present in the diet, and *n_i_* is the relative abundance of that species in the environment. Positive values show a preference for that species, while negative values show avoidance of that species. Values equal to zero show a selection of that species relative to their abundance in the environment (Cooper et al., 1999; Ivlev, 1961).

## 3 RESULTS

### 3.1 Prey densities

The mean base density for each of the four large herbivorous prey species seen during game census counts in the study area are shown in Table 1. A half‐normal cosine model was used in the DISTANCE calculations, showing lower Akaike's Information Criterion (AIC) scores and % coefficient of variation (% CV) for each species' model fit (Table 2). Gemsbok had a mean density of 1.229/km^−2^ (±0.50) with a detection probability of 0.67 and an effective stripe width (ESW) of 645.95 m. Springbok had a slightly higher mean density of 1.352/km^−2^ (±0.48) with a detection probability of 0.60 and an ESW of 511.66 m. Ostrich had a density of 0.648/km^−2^ (±0.23) with a detection probability of 0.65 and an ESW of 918.99 m. Kudu proved the lowest and most inconsistently recorded dataset with a density of 0.343/km^−2^ (±0.0). The detection probability for kudu was 1.00 with an ESW of 211.00 m.

**TABLE 1:** Monthly mean prey group densities (km^−2^) with standard error (±*SE*) and degree of freedom (*df*) as calculated by DISTANCE software

Month	Prey Species			
	Gemsbok	Springbok	Ostrich	Kudu^a^
	km^−2^ (*SE*) (*df*)	km^−2^ (*SE*) (*df*)	km^−2^ (*SE*) (*df*)	km^−2^ (*SE*) (*df*)
Jan	0.736 ± 0.16 (3)	2.100 ± 0.71 (3)	0.252 ± 0.14 (3)	0.03 ± 0 –
Feb	1.837 ± 0.49 (3)	1.230 ± 0.31 (3)	0.453 ± 0.07 (3)	–
Mar	0.805 ± 0.17 (3)	1.144 ± 0.37 (3)	0.409 ± 0.19 (3)	–
Apr	1.816 ± 0.50 (3)	1.344 ± 0.39 (3)	0.238 ± 0.26 (3)	0.01 ± 0 –
May	0.127 ± 0.60 (2)	3.606 ± 1.30 (2)	0.868 ± 0.20 (2)	–
Jun	1.672 ± 0.43 (2)	0.420 ± 0.30 (2)	1.070 ± 0.29 (2)	–
Jul	0.518 ± 0.38 (2)	0.713 ± 0.17 (2)	0.814 ± 0.09 (2)	0.03 ± 0 –
Aug	1.535 ± 0.38 (2)	0.260 ± 0.36 (2)	0.404 ± 0.17 (2)	–
Sep	0.743 ± 0.20 (2)	0.230 ± 0.19 (2)	0.626 ± 0.34 (2)	–
Oct	3.456 ± 1.26 (3)	1.539 ± 0.37 (2)	0.377 ± 0.35 (2)	–
Nov	0.476 ± 0.35 (3)	0.669 ± 0.14 (2)	1.022 ± 0.26 (2)	–
Dec	1.037 ± 0.17 (2)	2.972 ± 1.18 (2)	1.244 ± 0.37 (2)	0.05 ± 0 –
µ	1.229 ± 0.50	1.352 ± 0.48	0.648 ± 0.23	0.343 ± 0.0

^a^ Greater Kudu sightings were not consistent enough for accurate calculations from the sample size.

**TABLE 2:** Density of large herbivorous prey species (km^−2^) calculated by DISTANCE software

Species	Density (±*SE*)	AIC	% CV
Gemsbok	1.229 (±0.50)	60.83	37
Springbok	1.352 (±0.48)	58.93	64
Ostrich	0.648 (±0.23)	63.80	40
Kudu	0.343 (±0.00)	49.06	34

Note

The half‐normal cosine model was used, Akaike's Information Criterion (AIC) score and the % coefficient of variation (% CV) show model fit.

### 3.2 Spotted hyaena scat sampling

A total of 146 spotted hyaena scats were collected during the study both in the study area (*n* = 128) and on neighboring properties (*n* = 17), representing a total of 240 identified prey items and eight identified species (Table 3). Because our samples were not collected seasonally, seasonal diet variations were not investigated. Of these samples, two feces (1.4%) contained unidentifiable prey items, 83 (57%) contained only one species of prey item, 58 (40%) contained two or more species of prey items, and 8% of all samples contained some form of vegetation matter. Gemsbok hairs were identified in 92 (63.01%) instances of all sampled scats. Springbok hairs were identified in 64 (43.83%) instances of all sampled scats. Ostrich, verified by their distinctive feathers and egg fragments as a food source, were identified in 2 (1.37%) instances of all sampled scats. Kudu hairs were identified in 6 (4.11%) instances of all sampled scats. The only recorded instance (0.68%) of livestock presence in a scat sample was domestic horse hairs identified in one scat.

**TABLE 3:** Prey items found in 146 scat samples

Prey identified	Number of instances	% Presence within scats
Gemsbok	92	63.01
Springbok	64	43.83
Kudu	6	4.11
Hyaena^a^	21	14.38
Aardwolf	1	0.68
Klipspringer	5	3.45
Livestock^b^	1	0.68
Small mammal^c^	5	3.42
Felid^d^	1	0.68
Ostrich egg	1	0.68
Ostrich feather	1	0.68
Bone fragments	27	18.49
Hoof fragments	2	1.37
Vegetation/grass	13	8.90
Totals	240 items identified	

^a^ Hyaena samples found within scat are assumed to result from self and social grooming (Kruuk, 1972) as opposed to cannibalism.

^b^ The one livestock sample identified was domestic horse.

^c^ Small mammals are defined as mammalian vertebrates under 3 kg adult weight.

^d^ Felid defines any remains from a felid species. The one recorded instance was a felid claw.

### 3.3 Ivlev's Electivity Index

The Ivlev's Electivity Index results for each prey species against their relative abundance in the study area are shown in Table 4. Significance was checked using the 2‐tailed Fisher's Exact Test (Agresti, 1992), (Table 4). Gemsbok had the closest score to 0 (selection relative to abundance) of −0.320. Springbok showed a lower selection of −0.511, and kudu was the second lowest selection of −0.786. Ostrich was the most avoided prey item at −0.961.

**TABLE 4:** Results of the Ivlev's Electivity Index for each of the four sampled large herbivorous prey

Prey species	Ivlev's electivity index	Fisher's exact test
		2‐tailed *p*‐value
Gemsbok	*E* = −0.320	*p* =.267989
Springbok	*E* = −0.511	*p* =.000935
Ostrich	*E* = −0.961	*p* = 1.567507
Kudu	*E* = −0.786	*p* =.000108

Note

Fisher's exact test was used to test for the 2‐tailed *p*‐value of each prey species' results (Agresti, 1992).

## 4 DISCUSSION

The diet investigation of spotted hyaena in the south Namib Desert showed a higher tendency of finding gemsbok prey in scat than any other large herbivorous prey species present. Electivity Index results showed gemsbok to have the highest selectivity of the four sampled large prey species; however, this was still a negative value suggesting an avoidance relative to their abundance. Gemsbok and springbok were the highest recorded prey items across the scats collected, suggesting that large herbivorous prey is in general an important food source. However, the hypothesis of the most numerous large prey species being the most identified item in the scats was not supported as springbok showed a higher mean density within the environment compared with gemsbok. Although springbok were selected more as a preferred prey item by spotted hyaenas in central Etosha Park over larger‐bodied prey (Trinkel, 2009), this was reflected by smaller hunting group sizes targeting smaller prey. Reasoning could be due to the lower food reward gain of pursuing smaller‐bodied prey (as mentioned with Thomson's gazelle by Cooper et al., 1999) by multiple hyaenas. Investigations into the population densities of spotted hyaena within the study area are thus needed to ascertain a hunting group size and if this determines the size of prey being targeted.

Kudu were less frequently encountered both during the game census counts and in representation within scats. This low encounter rate of kudu could be contributed to lingering after‐effects of rabies outbreaks within the kudu populations (Mansfield et al. 2006) leading to few sightings and encounters. Some studies (Wentworth et al., 2011) have mentioned a relative avoidance of kudu, while others (Hayward, 2006; Trinkel, 2009) show a preference for kudu. The low preference for ostrich as a food item is well supported by other studies (Hayward, 2006). While ostrich does occur in other spotted hyaena diet assessments, those occurrences are limited to low recordings (1 instance each: Gasaway et al., 1991; Kruuk, 1972; Tilson et al., 1980). Although livestock densities were not tested for electivity the same way wildlife prey species were in this study, the low presence of livestock within the sampled spotted hyaena scat suggests a dietary breadth with a higher focus on wildlife rather than livestock species. This scenario can likely be attributed to direct and effective livestock husbandry techniques, as described by Ogada et al. (2003), utilized on the neighboring communal and commercial livestock properties to reduce predator depredation.

From this study and the electivity test, we can conclude that all large herbivorous prey are avoided by spotted hyaenas in relation to their density in the environment. Spotted hyaena in the Namib Desert have a higher occurrence of gemsbok in their diets over other large herbivorous prey, which is supported by previous findings of Namib hyaenas by Tilson et al. (1980). However, this still represents an avoidance in relation to their abundance in the environment and cannot be determined as a direct preferred selection. This upholds the consensus by Henschel and Tilson (1988) that Namib hyaenas do not depress prey populations. As only larger prey species were sampled by the employed census method, nonsampled smaller‐bodied prey species, although recorded within the diet (e.g., klipspringer and unidentified small mammal hairs), were not assessed and therefore could constitute a higher selection and possible preference over large‐bodied prey. Further investigations into medium and small prey species within the study area should be undertaken to determine a finer‐scale dietary breadth of Namib spotted hyaenas. Another factor to consider for further investigation is the seasonal variances in prey abundance compared with seasonally collected scat samples, which may reveal seasonal based, but not necessarily overall preferences for certain prey species.

## CONFLICT OF INTEREST

There are no competing interests concerning this manuscript and data between the listed authors, institutions, or any third parties.

## AUTHOR CONTRIBUTIONS

**Karl Sebastian Moritz Fester:** Conceptualization (lead); formal analysis (lead); investigation (equal); methodology (equal); project administration (lead); resources (lead); supervision (lead); validation (lead); visualization (equal); writing–original draft (lead). **Georgina Hockings:** Investigation (equal); methodology (equal); visualization (equal); writing–review and editing (lead). **Rudie Jansen van Vuuren:** Funding acquisition (equal); writing–review and editing (supporting). **Marlice van Vuuren:** Funding acquisition (equal); writing–review and editing (supporting).

## Data Availability

The data collected for this study have not utilized shared data. The data are available at: https://datadryad.org/stash/share/efOOSskihr8TWlhKVrcZnbpn‐ABUEgXegN875pVPh3w.
